# Catalytic Upgrading of Lignocellulosic Biomass Sugars Toward Biofuel 5-Ethoxymethylfurfural

**DOI:** 10.3389/fchem.2021.831102

**Published:** 2022-01-31

**Authors:** Xiaofang Liu, Dayong Yu, Hangyu Luo, Can Li

**Affiliations:** Guizhou Provincial Key Laboratory for Rare Animal and Economic Insects of the Mountainous Region, College of Biology and Environmental Engineering, Guiyang University, Guiyang, China

**Keywords:** lignocellulosic biomass, biorefinery, high-value chemicals, biofuels, 5-ethoxymethylfurfural

## Abstract

The conversion of biomass into high-value chemicals through biorefineries is a requirement for sustainable development. Lignocellulosic biomass (LCB) contains polysaccharides and aromatic polymers and is one of the important raw materials for biorefineries. Hexose and pentose sugars can be obtained from LCB by effective pretreatment methods, and further converted into high-value chemicals and biofuels, such as 5-hydroxymethylfurfural (HMF), levulinic acid (LA), γ-valerolactone (GVL), ethyl levulinate (EL), and 5-ethoxymethylfurfural (EMF). Among these biofuels, EMF has a high cetane number and superior oxidation stability. This mini-review summarizes the mechanism of several important processes of EMF production from LCB-derived sugars and the research progress of acid catalysts used in this reaction in recent years. The influence of the properties and structures of mono- and bi-functional acid catalysts on the selectivity of EMF from glucose were discussed, and the effect of reaction conditions on the yield of EMF was also introduced.

## Introduction

Extensive use of fossil fuels has caused energy depletion and serious environmental problems (e.g., greenhouse effect and acid rain). It is urgent to develop green renewable energy to replace fossil fuels for a better living environment (Li et al., 2017; Li et al., 2020; Pan et al., 2020). Lignocellulosic biomass (LCB) is a typical renewable energy with an annual global output of approximately 12 billion tons (Abraham et al., 2020). It is mainly composed of a layer of firm lignin-wrapped cellulose and hemicellulose components (Bhatia et al., 2020). Among them, cellulose is a biopolymer linking massive glucose units *via β*-1,4-glycosidic bonds, accounting for 38–50 wt% of LCB (Somerville et al., 2010). Thus, a large amount of glucose can be obtained by hydrolyzing cellulose. There were many researchers focused on the conversion of glucose to high value-added chemicals. Through various catalytic reactions such as dehydration, hydrogenation, hydrolysis, alcoholysis, and etherification, glucose can be turned into high value-added fuels and fine chemicals [e.g., 5-hydroxymethylfurfural (HMF), 5-ethoxymethylfurfural (EMF), levulinic acid (LA), and ethyl levulinate (EL)] (Rackemann and Doherty, 2011; Yang et al., 2012; Climent et al., 2014; Yang et al., 2019).

Furan derivatives like furfural, furfuryl alcohol, HMF, EMF, and 2,5-dimethylfuran have shown great potential in the formation of fine chemicals and alternative fossil fuels (Tong et al., 2010; Liu et al., 2021). Among these furan derivatives, EMF has the advantages for instance a higher boiling point (235°C), superior energy density (30.3 MJ/L) compare with ethanol (23.5 MJ/L), and low flash point (ca. 110°C) (Corma et al., 2007). Therefore, it has been considered one of the excellent choices of fuel additives in the future (Li et al., 2016). When 17 wt% EMF was used as an additive that mixes with fuel in a fuel engine, the engine could run stably and release fewer harmful particles and sulfides (with a 16% reduction in soot) (Mascal and Nikitin, 2008). In addition, EMF has also be used as a reaction substrate for the synthesis of various industrially significant chemicals, such as 5-ethoxymethylfurfuryl alcohol, 2,5-diethoxymethylfuran, and cyclopentenone (Ras et al., 2009; Ras et al., 2010; Bredihhin et al., 2016).

Generally, EMF can be transformed from HMF and ketose (e.g., fructose, inulin, and sucrose) with a satisfactory yield (ca. 70–90%) (Bredihhin et al., 2013; Dai et al., 2019; Hafizi et al., 2020). Yet, the industrial-scale production of EMF was limited by these high-priced feedstocks. For example, the price of HMF and fructose in Sigma-Aldrich is 12,634 and 205 EUR per kilogram, respectively. However, glucose has a lower price (88 EUR per kilogram in Sigma-Aldrich), which is reasonable to convert glucose into EMF (187 EUR per gram in Sigma-Aldrich). Moreover, the large amount of glucose can be obtained from cheap LCB, which is also a choice for economic and environmental development. At present, relevant reviews have summarized the use of various types of catalysts to convert different raw materials into EMF (Chen B. et al., 2020; Yu et al., 2021). But almost no review focused on the mechanism of EMF synthesis from glucose to EMF. Hereby, this mini-review introduces the paths and mechanisms of producing EMF from LCB derivatives, with a focus on challenges of the conversion of glucose to EMF. The aim is to provide a feasibility method for maximizing the conversion of LCB into EMF.

## The Preparation of EMF From LCB-Derived Sugars

### The Synthesis Routes

**FIGURE 1:**
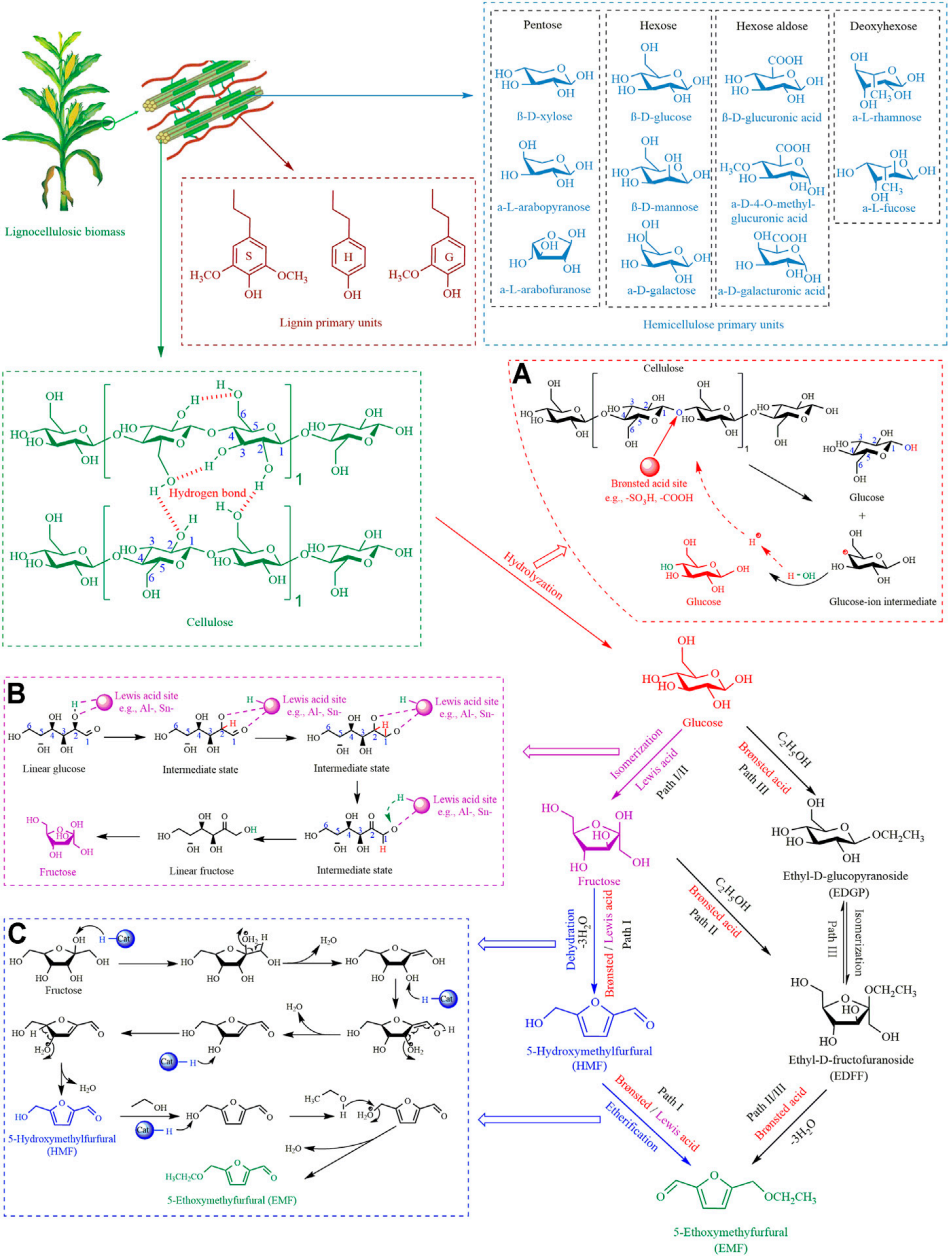
The conversion paths of LCB to EMF. **(A)** Cellulose hydrolysis by Brønsted acid, **(B)** glucose isomerization by Lewis acid, **(C)** the conversion of fructose to EMF.

EMF can be obtained from glucose or cellulose *via* multi-step chemical conversion (Zheng et al., 2021). There are three paths to synthesize EMF from glucose (Figure 1). The mainstream Path I uses glucose as the starting material, which is isomerized to produce fructose, then HMF is obtained through fructose dehydration (-3H_2_O), and finally, HMF is etherified to EMF (Chen et al., 2019). The most important step in this path is the isomerization of glucose, which usually requires the participation of Lewis acid (Lew et al., 2012). There are two other secondary paths with ethyl-D-fructofuranoside (EDFF) as an intermediate transit. Path Ⅱ is that fructose reacts with ethanol in acidic solution to form EDFF, which is then dehydrated (-3H_2_O) to produce EMF (Zhang et al., 2018). Path Ⅲ is glucose and ethanol to generate ethyl-D-glucopyranoside (EDGP) in an acid medium, then isomerized to EDFF, finally dehydrated (-3H_2_O) to obtain EMF (Zheng et al., 2021). Currently, most EMF is obtained through Path I for the following reasons:

(ⅰ) Compare Path I and Path Ⅱ. The difference is that fructose is more likely to be converted into HMF (Path I) or EDFF (Path Ⅱ). It has been found that fructose was inclined to be dehydrated to form HMF (Path I) rather than etherified with ethanol to form EDFF (Path Ⅱ) when Brønsted acid is present (Xiang et al., 2017).

(ⅱ) Compare Path I and Path Ⅲ. Glucose is usually isomerized to fructose when Brønsted acid and Lewis acid are present at the same time (He et al., 2022). When there is only Brønsted acid in the system, although the DFT calculation results show that the highest energy barriers required for Path I (17.7 kcal/mol) and Ⅱ (20.8 kcal/mol) are similar, the thermodynamic reaction is more favorable for Path I (Wang et al., 2021). And the intermediate EDGP in Path Ⅲ is difficult to continue further conversion.

### The Synthesis Mechanism

The conversion of cellulose to EMF requires multiple reaction processes, namely cascade reactions. A detailed description of the synthesis mechanism of each step in Path I is shown in Figure 1.

Cellulose has a condensed structure (Figure 1), and is a high molecular polymer connected by *β*-1,4-glycosidic bonds and axial hydrogen bonds between numerous glucose monomers (Shrotri et al., 2018). Therefore, the hydrolysis of cellulose in the first step of Path I is a major obstacle that needs to be overcome. Many studies have shown that Brønsted acid can destroy the *β*-1,4-glycosidic bonds of cellulose (Zeng and Pan, 2020). As shown in Figure 1A, firstly, the oxygen atom of the β-1,4-glycosidic bond is attacked by the proton of the Brønsted acid site. Then the C-O bond between the two glucose molecules is broken for releasing glucose and glucose-ion intermediate. Finally, the hydroxyl group from water binds to the exposed carbon of glucose-ion intermediate to form glucose. And the free protons from water participate in the next hydrolysis reaction.

The second step in Path I, the isomerization of glucose into fructose, is the most important step in determining the yield of EMF. Many studies have indicated that glucose transforms into fructose *via* Lewis acid sites (Li et al., 2014; Rajabbeigi et al., 2014). As shown in Figure 1B, the C_1_-O_5_ bond of glucose is broken by Lewis acid and forms a linear glucose molecule. The oxygen atoms of C_1_ and C_2_ on linear glucose coordinate with the Lewis acid center. Subsequently, the hydrogen on C_2_ is transferred to C_1_, which realizes the aldehyde-ketone conversion between C_1_ and C_2_ to form linear fructose. Finally, the oxygen of C_2_ is linked with C_5_ to form a fructose molecule by C-C bond.

The third step is that fructose generates HMF by dehydration of three H_2_O molecules under acidic conditions. Firstly, the hydroxyl group on C_2_ is protonated to release the first H_2_O, and C=C is formed between C_1_ and C_2_. Then, the hydroxyl group on C_3_ is protonated to release the second H_2_O. Meanwhile, the C=C bond between C_1_ and C_2_ is broken, the aldehyde group is formed at the C_1_, and C=C is formed between C_2_ and C_3_. Finally, the hydroxyl group on C_4_ is protonated to release the third H_2_O, and C=C is formed between C_4_ and C_5_ to get HMF. After that, HMF is etherified to EMF with ethanol existence (Figure 1C).

## The Factors Impacting the Yield of EMF From LCB-Derived Sugars

Many LCB-derived sugars and compounds have been used to convert into EMF, such as cellulose, cellobiose, and glucose. The EMF yield from these substrates has displayed the order of glucose > cellobiose > cellulose > LCB (Li et al., 2016; Guo et al., 2018). In general, only moderate or low EMF yields can be obtained from these raw materials which are due to the different number of reaction steps. For example, HMF as a feedstock (high EMF yield) just needs one step, but cellulose (low EMF yield) needs four steps. Meanwhile, the lengthy chemical reaction process increased more by-products or humins (Zheng et al., 2021). Therefore, many studies were devoted to developing more efficient catalytic systems, which can obtain more satisfactory EMF yields from glucose or glucose-based carbohydrates (Guo et al., 2017; Guo et al., 2018; Karnjanakom et al., 2020; He et al., 2022). Some catalysts and reaction conditions for obtaining EMF from LCB-derived sugars were summarized in Table 1.

**TABLE 1:** EMF from LCB-derived sugars *via* different catalysts and reaction systems.

Entry	Feedstock	Catalyst	Brønsted acid	Lewis acid	Solvent	Reaction conditions	Yield/%	Ref.
1	Glucose	H_2_SO_4_	-SO_3_H	—	Ethanol	200°C, 90 min	7.5	Xu et al. (2017)
2	Glucose	[BMIM][HSO_4_]	-SO_3_H	—	Ethanol	130°C, 20 min	8.0	Guo et al. (2017)
3	Glucose	AlCl_3_	—	Al-	Ethanol	100°C, 11 h	38.4	Liu et al. (2013)
4	Corn Stover	USY	Al-O(H)-Si	Al-	Ethanol/THF (v/v = 1:1)	168°C, 2.9 h	21.8	Chen et al. (2019)
5	Glucose	DeAl-H-β	Al-O(H)-Si	Al-	Ethanol	125°C, 10 h	41.0	Li et al. (2016)
6	Glucose	MFI-Sn/Al	Al-O(H)-Si	Sn-/Al-	Ethanol	140°C, 9 h	44.0	Bai et al. (2018)
7	Glucose	BFC-3	-SO_3_H	Cr-	Ethanol/THF (v/v = 3:2)	100°C, 10 h	48.1	Chen et al. (2020a)
8	Cellobiose	BFC-3	-SO_3_H	Cr-	Ethanol/THF (v/v = 3:2)	100°C, 10 h	37.1	Chen et al. (2020b)
9	Glucose	Zr-Sn-Fe-Al-O-S	-SO_3_H	Zr-/Sn-/Fe-/Al-	Ethanol	160°C, 12 h	0.7	He et al. (2022)
10	Glucose	Zr-Sn-Fe-Al-O-S	-SO_3_H	Zr-/Sn-/Fe-/Al-	Ethanol/DMSO (v/v = 9:1)	160°C, 4 h	3.9	He et al. (2022)
11	Glucose	Zr-Sn-Fe-Al-O-S	-SO_3_H	Zr-/Sn-/Fe-/Al-	Ethanol/DMSO (v/v = 9:1)	160°C, 12 h	7.9	He et al. (2022)
12	Glucose	Zr-Sn-Fe-Al-O-S	-SO_3_H	Zr-/Sn-/Fe-/Al-	Ethanol/DMSO (v/v = 3:1)	160°C, 12 h	14.3	He et al. (2022)
13	Glucose	Zr-Sn-Fe-Al-O-S	-SO_3_H	Zr-/Sn-/Fe-/Al-	Ethanol/DMSO (v/v = 1:1)	160°C, 12 h	33.1	He et al. (2022)
14	Glucose	Zr-Sn-Fe-Al-O-S	-SO_3_H	Zr-/Sn-/Fe-/Al-	Ethanol/DMSO (v/v = 1:3)	160°C, 12 h	12.4	He et al. (2022)
15	Glucose	Zr-Sn-Fe-Al-O-S	-SO_3_H	Zr-/Sn-/Fe-/Al-	Ethanol/DMSO (v/v = 1:1)	140°C, 12 h	18.8	He et al. (2022)
16	Glucose	Zn-S-C	-SO_3_H	Zn-	Ethanol/THF (v/v = 1:1)	Ultrasonic system: 98°C, 47 min	80.9	Karnjanakom et al. (2020)
17	Cellobiose	Zn-S-C	-SO_3_H	Zn-	Ethanol/THF (v/v = 1:1)	Ultrasonic system: 98°C, 47 min	74.6	Karnjanakom et al. (2020)
18	Glucose	Zn-SO_3_H-GR-carbon	-SO_3_H	Zn-	Ethanol/THF (v/v = 1:2)	Ultrasonic system: 106°C, 72 min	86.3	Karnjanakom and Maneechakr, (2019a)
19	Glucose	Al-SC	-SO_3_H	Al-	Ethanol/THF (v/v = 1:1)	Ultrasonic system: 106°C, 72 min	84.4	Karnjanakom and Maneechakr, (2019b)
20	Glucose	Zn-SC	-SO_3_H	Zn-	Ethanol/THF (v/v = 1:1)	Ultrasonic system: 106°C, 72 min	85.1	Karnjanakom and Maneechakr, (2019b)

### Monofunctional Acid Catalysts

Currently, many monofunctional (Brønsted or Lewis) acid catalysts are designed to catalyze the synthesis of EMF from glucose, including H_2_SO_4_ (Xu et al., 2017), metal salts (Liu et al., 2013), SO_3_H-based catalyst (Liu and Zhang, 2013), and ionic liquid (Guo et al., 2017). From the perspective of the synthesis routes, theoretically, when only Brønsted acid exists, using glucose as substrates hardly produces EMF. Yet, many studies had found that in the presence of Brønsted acid, a spot of EMF could be detected using glucose (7.46% yield), cellobiose (19.8% yield), and cellulose (3.05% yield) as raw materials (De et al., 2012; Guo et al., 2017; Xu et al., 2017). One possible reason for this is that glucose formed a bit intermediate 3-deoxyglucosone in Brønsted acid, which is then dehydrated to form HMF, and finally etherified to EMF (Jadhav et al., 2011; Jadhav et al., 2012). In addition, Brønsted acid and protonated ethanol ([C_2_H_5_OH_2_]^+^) can open the ring of glucose to form intermediate 1,2-enediol then isomerizes to fructose, which makes it possible to produce EMF in the next step (Guo et al., 2017; Wang et al., 2021). When there is only Lewis acid in the system, a moderate EMF yield (10–40%) can be obtained from glucose (Dutta et al., 2012; Liu et al., 2013; Tan et al., 2017). In the presence of a single Lewis acid, the possible reason for the failure to obtain high EMF yield is that the Lewis acid cannot provide H^+^, resulting in the low [C_2_H_5_OH_2_]^+^ concentration in the system which limits the fructose dehydration and subsequent etherification steps. Meanwhile, the EMF yields obtained by catalyzing glucose with different types of metal salts were quite different. Such as metal chlorides AlCl_3_ and CrCl_3_ could obtain 11.2 and 15.2% EMF yields, respectively. However, with metal sulfates Al_2_(SO_4_)_3_, CuSO_4_, Fe_2_(SO_4_)_3_, and Cr_2_(SO_4_)_3_ as catalysts, the reaction system hardly detected EMF, but more EDGP (ca. 80% yield) was detected (Yu et al., 2017). Thus, the metal chloride is more conducive to the isomerization of glucose, while the metal sulfate is more inclined to promote the etherification of glucose. Overall, the monofunctional acid catalysts cannot obtain satisfactory EMF yield from glucose. Whereas, the developed bifunctional acid catalysts with Brønsted-Lewis acids can obtain high EMF yield from glucose.

### Bifunctional Acid Catalysts

Generally, zeolite molecular sieve catalysts contain Brønsted acid species Al-O(H)-Si (framework four-coordinate aluminum), and Lewis acid species Al- (framework three-coordinate aluminum) can be obtained after high-temperature dealumination (Xin et al., 2019). For example, ultra-stable Y zeolite (USY) and β zeolite (H-*β*) after high-temperature dealumination were used to catalyze the synthesis of EMF from glucose and obtained 39.5 and 41% EMF yields, respectively (Li et al., 2016; Zheng et al., 2021). In addition, zeolite can also be modified to obtain better EMF yield. Introducing Lewis acid species Sn- and Al- into zeolite to obtain MFI-Sn/Al (Bai et al., 2018) or simultaneously introduce H_4_ [Si(W_3_O_10_)_4_] and SnCl_4_ (Brønsted-Lewis acids) into zeolite to obtain SBA-15 (Srinivasa Rao et al., 2020). These catalysts could obtain EMF with medium yield from glucose. A soft template HIPE was utilized to support the sulfonic acid group and Cr^3+^ to synthesize BFC-3 catalyst, which could be used to catalyze glucose and cellobiose and obtain 48.1 and 37.1% EMF yields, respectively (Chen et al., 2020a). Furthermore, glycerol and glucose were sulfonated into carbon spheres, then introduced into Zn- to prepare Zn-SO_3_H-GR-carbon (Karnjanakom and Maneechakr, 2019a) and Zn-S-C (Karnjanakom et al., 2020). Both of them can obtain amazing EMF yields from glucose (86.3 and 80.9%).

Bifunctional acid catalysts have great differences in catalytic performance. Using the same Brønsted acid and different Lewis acids to prepare various catalysts, the yields of EMF obtained from glucose were different. For example, the sulfonated carbon (SC) was doped with different metal species (Zn-, Al-, and Ni-), which exhibits different catalytic performances (Karnjanakom and Maneechakr, 2019b). When EMF was selectively produced from glucose, Zn-SC, Al-SC, and Ni-SC provided yields of 85.1, 84.4, and 32.8%, respectively. The reason for the difference is that the acidity provided by specie Ni- is lower than Zn- and Al-. A recent study also confirmed that the type of Lewis acids affects the yield of EMF (He et al., 2022). Meanwhile, this study found that the performance of the catalyst was also affected by the number of metal species in it. Specifically, the more types of metals contained in the catalyst, the better the catalytic efficiency. In addition, choosing a suitable ratio of Brønsted/Lewis acid can improve the selectivity of EMF (Srinivasa Rao et al., 2020). For the ratio of strong/weak acid, when the weak acid accounts for more, it is harmful to glucose isomerization, fructose dehydration, and HMF etherification, resulting in lower EMF selectivity. On the contrary, when more strong acids are present in the system, the generated EMF can be turned into EL by a ring-opening reaction or converted into humins (He et al., 2022). Therefore, the ratio of Brønsted/Lewis and strong/weak acids in the bifunctional acid catalysts are also critical for obtaining EMF from glucose.

### Reaction Conditions

The selectivity of EMF is also affected by the reaction conditions, such as temperature, time, ultrasound, and co-solvent.

Obtaining EMF from glucose usually requires a higher temperature and longer reaction time (Srinivasa Rao et al., 2020; Wang et al., 2021). However, continuously increasing the reaction temperature and time leads to the decrease in the yield of EMF, which is due to the unstable EMF and easily converted to EL under high temperature and long time (Zheng et al., 2021). During the conversion of glucose to EMF, water may be produced due to dehydration and etherification, which makes the hydrolysis of HMF into LA inevitable in this system (Wang et al., 2021). Since the polar co-solvent limits the conversion of HMF to LA (Morales et al., 2017), such as dimethyl sulfoxide (DMSO), tetrahydrofuran (THF), and GVL, many studies add co-solvents to this system, which significantly inhibited the production of EL (Yu et al., 2018). The amount of co-solvent also affects the yield of EMF. With the increase of co-solvent ratio, the yield of EMF first increased and then decreased, while the yield of EL continued to decrease and HMF continued to increase (He et al., 2022). The increase of EMF can be attributed to the inhibition of the conversion of EMF to EL. Then adding too much co-solvent can reduce the amount of EMF, which is attributed to the decrease of ethanol content in the system to limit the etherification of HMF into EMF (Chen et al., 2020b). Besides, several studies have shown that ultrasonic assistance can form cavitation bubbles in the system and promote bond breakage, which can promote the reaction to a certain extent (Karnjanakom and Maneechakr, 2019b). The ultrasound assistance can greatly reduce the requirement of temperature and time from glucose to high yield EMF, such as 98°C for 47 min obtained 80.9% yield (Karnjanakom et al., 2020) and 106°C for 72 min obtained 86.3% yield (Karnjanakom and Maneechakr, 2019a). Therefore, EMF can be generated rapidly under mild conditions.

## Conclusion

The richness, versatility, and accessibility of LCB are the reasons for its advantages in the field of sustainable energy conversion. The mechanisms and technologies of EMF production from LCB-derived sugars in recent years were reviewed. These studies aim to develop more efficient catalysts and reaction systems to increase the yield of EMF.

Glucose as a typical LCB-derived sugar is used to synthesize EMF. It is mainly through path I (Figure 1) to synthesize EMF. In general, it shows low EMF yield when used monofunctional acid catalysts. The key to this problem is attributed to the glucose isomerization step (corresponding to Lewis acid) and low concentration of [C_2_H_5_OH_2_]^+^ (corresponding to Brønsted acid). Yet, the developed bifunctional (Brønsted-Lewis) acid catalysts can effectively solve this problem, which can obtain satisfactory EMF yields from glucose. Meanwhile, the species of Lewis acids, ratio of Brønsted/Lewis acids, and ratio of strong/weak acids in the bifunctional acid catalysts have decisive effects on EMF yield. In addition, the optimization of reaction conditions has also made efforts in EMF yield. The suitable time, temperature, and a certain concentration of co-solvent can provide upside space for the selectivity of EMF.

## Perspectives

Although there are some technological breakthroughs in obtaining high EMF yield from glucose, high yield EMF has not been found directly from LCB. However, studies based on glucose can provide feasible strategies for direct conversion of LCB to obtain high EMF in the future. Firstly, the bifunctional acid solid catalysts were given priority in the choice of catalysts, and the catalysts can adjust the type and quantity of acid. Secondly, it is also crucial to select appropriate co-solvents and reaction conditions. Although the ultrasound-assisted method showed excellent effects, it is not suitable for large-scale industries. Therefore, it is of great significance to develop an efficient catalyst strategy to convert LCB into EMF under mild conditions.
